# FusedFCR: A Fused Forward Continuation-Ratio model for marker selection along cell-fate trajectories

**DOI:** 10.64898/2026.07.20.739649

**Published:** 2026-08-21

**Authors:** Chloe Mattila, Anirban Chakraborty, Peggi Angel, Sha Cao, Kalyani Sonawane, Elizabeth Hill, Dongjun Chung, Brian Neelon, Souvik Seal

## Abstract

Time-course single-cell RNA sequencing (scRNA-seq) data collected across ordered stages provide population-level snapshots of differentiation, disease progression, and aging. Supervised pseudotime methods use observed stage labels to reconstruct continuous progression but generally do not identify marker genes associated with changes from one stage to the next. Unsupervised pseudotime-based marker selection methods infer latent trajectories directly from expression data and identify trajectory-associated genes, but do not explicitly link these associations to the observed stages. We propose FusedFCR, a regularized forward continuation-ratio model that represents cellular progression through a sequence of conditional transitions across ordered stages. FusedFCR combines a lasso penalty for gene selection with a fusion penalty that encourages similar effects across adjacent transitions while allowing transient and direction-changing associations. The resulting transition-specific coefficients support interpretable gene selection and a continuous pseudotime-like projection anchored to the observed developmental stages. In simulations, FusedFCR accurately recovers gene-effect trajectories and improved predictive performance relative to alternative methods. Applied to one mouse and three human datasets (mouse pancreatic beta-cells, human extravillous trophoblast, human induced pluripotent stem cell derived astrocytes, and human endometrial cells during the secretory phase), FusedFCR identifies biologically interpretable genes associated with distinct developmental transitions. Gene set enrichment analysis further reveals stage-specific pathway activity consistent with known developmental biology, while held-out stage-classification accuracy was competitive or superior across both datasets. Together, these results show that FusedFCR complements pseudotemporal ordering by identifying which molecular programs change and when those changes emerge along the developmental trajectory. An accompanying R package is available on GitHub.

## Full Text Availability

The license terms selected by the author(s) for this preprint version do not permit archiving in PMC. The full text is available from the preprint server.

