# Supplementary material for: FusedFCR: A Fused Forward Continuation-Ratio model for marker selection along cell-fate trajectories
Source: bioRxiv. 2026 Jul 24:2026.07.20.739649. Preprint. [Version 1] doi: 10.64898/2026.07.20.739649 (PMC13419772; doi:10.64898/2026.07.20.739649)
Supplement: Supplementary file 1 [file media-1.pdf]

FusedFCR Supplementary Material

Chloe Mattila†1, Anirban Chakraborty†1, Peggi Angel[2], Sha Cao[3], Kalyani Sonnawane[1], Elizabeth Hill[1], Dongjun Chung[4], Brian Neelon[1], and Souvik Seal*1

[1]Department of Public Health Sciences, Medical University of South Carolina, Charleston, SC, USA

[2]Department of Pharmacology and Immunology, Medical University of South Carolina, Charleston, SC, USA

[3]Department of Biomedical Engineering, Oregon Health & Science University, Portland, OR, USA

[4]Department of Biomedical Informatics, The Ohio State University, Columbus, Ohio, USA

1 Derivation of the unconditional probabilities

We start with the definition of π_{ik} which is given by,

$$\pi_{ik} = P(Y_i = k \mid Y_i \geq k) = 1 - P(Y_i > k | Y_i \geq k). \tag{1.1}$$

In addition, we note that for any set A that is a subset of B, i.e., $A \subset B$, we can always write

$$P(A) = P(A \cap B),$$

†These authors contributed equally to this work.

*Corresponding author: sealso@musc.edu

since both of A and B occur simultaneously only when A occurs. Using the above identity and applying equation (1.1) recursion, we can derive the unconditional probability $P(Y_i = k)\ \forall k < K$ as,

$$\begin{aligned}
P(Y_i = k) &= P(Y_i = k, Y_i \geq k) \\
&= P(Y_i = k | Y_i \geq k) P(Y_i \geq k) \\
&= P(Y_i = k | Y_i \geq k) P(Y_i \geq k | Y_i \geq k-1) P(Y_i \geq k-1) \\
&= P(Y_i = k | Y_i \geq k)(1 - P(Y_i = k-1 | Y_i \geq k-1)) P(Y_i \geq k-1) \\
&= \pi_{ik}(1 - \pi_{ik-1}) P(Y_i \geq k-1) \\
&= \pi_{ik}(1 - \pi_{ik-1}) P(Y_i \geq k-1 | Y_i \geq k-2) P(Y_i \geq k-2) \\
&= \pi_{ik}(1 - \pi_{ik-1})(1 - \pi_{ik-2}) P(Y_i \geq k-2) \\
&\ \ \vdots \\
&= \pi_{ik}(1 - \pi_{ik-1})(1 - \pi_{ik-2}) \ldots P(Y_i \geq 1) \\
&= \pi_{ik} \prod_{u=1}^{k-1} (1 - \pi_{iu}),
\end{aligned} \tag{1.2}$$

since $\pi_{i1} = P(Y_i = 1) = 1 - P(Y_i \geq 1)$. Using the notation $p_{ik} = P(Y_i = k)$ from Section **??**, we can write,

$$\begin{aligned}
p_{i1} + p_{i2} &= \pi_{i1} + \pi_{i2}(1 - \pi_{i1}) = 1 - (1 - \pi_{i1})(1 - \pi_{i2}) \\
p_{i1} + p_{i2} + p_{i3} &= 1 - (1 - \pi_{i1})(1 - \pi_{i2}) + \pi_{i3} \prod_{u=1}^{2} (1 - \pi_{iu}) = 1 - \prod_{u=1}^{3} (1 - \pi_{iu}) \\
&\ \ \vdots \\
\sum_{u=1}^{K-1} p_{ik} &= 1 - \prod_{u=1}^{K-1} (1 - \pi_{iu}).
\end{aligned}$$

Using the above identities, it is straightforward to show that,

$$P(Y_i = K) = 1 - \sum_{u=1}^{K-1} p_{ik} = \prod_{u=1}^{K-1} (1 - \pi_{iu}). \tag{1.3}$$

Combining equations (1.2) and (1.3), we can express p_{ik} as,

$$p_{ik} = (\pi_{ik})^{\mathbb{I}(k<K)} \prod_{u=1}^{k} (1 - \pi_{iu}).$$

2 Numerical considerations

2.1 Stabilizing optimization and hyperparameter tuning

The performance of cross validation strategy outlined in Section 3 of the main manuscript depends on the number of observations in specific stages. To illustrate this let us define $n_k = \sum_{i=1}^{N} 1\{y_i = k\}$, i.e., the number of observations recorded at a specific stage k. In many biological applications, observations vary a lot across stages, and hence n_1 may be significantly different from n_2. As Adhikari et al. (2019)[1] have observed, when this happens, the objective function outlined in equation 2.6 of the main manuscript performs poorly in terms of prediction as well as feature selection. To avoid this numerical collapsing, we first modify the log-likelihood contribution as,

$$l^a(\{\alpha_{0k}, \boldsymbol{\beta}_k\}_{k=1,\ldots,K} | \mathbf{y}) = \sum_{k=1}^{K} \frac{1}{n_k} \sum_{i:i \in R(k)} \left[\mathbb{I}_{ik}(\alpha_{0k} + \boldsymbol{\beta}_k^T \mathbf{x}_i) - \log\left(1 + e^{\alpha_{0k} + \boldsymbol{\beta}_k^T \mathbf{x}_i}\right)\right].$$

For each of the $K - 1$ stages, we standardize the stage-specific total likelihood contribution by the number of recorded observations in that stage. This mitigates the effect of class imbalance. Consequently, we proceed with the new objective function $F^a_{\lambda_1,\lambda_2}(\Theta)$ which can be expressed as,

$$F^a_{\lambda_1,\lambda_2}(\Theta) = -l^a(\Theta \mid \mathbf{z}) + P_{\lambda_1,\lambda_2}(\boldsymbol{\beta}_1, \ldots . \boldsymbol{\beta}_p) \tag{2.1}$$

We proceed with the new fused lasso objective (2.1) for the subsequent parameter estimation steps of α_{0k} and $\boldsymbol{\beta}_k$'s.

2.2 Selection of the optimal learning rate

The score functions in Algorithm 1 in the main manuscript do not have a closed form. A Newtons first order method is generally used in optimizing α_{0k} and $\boldsymbol{\beta}_k$'s, i.e.,

$$\begin{aligned}
\alpha_{0k}^{(m+1)} &= \alpha_{0k}^{(m)} - \eta_m \nabla_{\alpha_{0m}} F^a_{\lambda_1,\lambda_2}(\Theta^{(m)}) \\
\tilde{\boldsymbol{\beta}}_k^{(m+1)} &= \boldsymbol{\beta}_1^{(m)} - \eta_m \nabla_{\boldsymbol{\beta}_k} l^a(\Theta^{(m)}) \\
\boldsymbol{\beta}_{\cdot j}^{(m+1)} &= \mathrm{prox}_{\lambda_1,\lambda_2,\eta_m}\left(\tilde{\boldsymbol{\beta}}_{\cdot j}^{(m+1)} - \eta_m \nabla_{\boldsymbol{\beta}_{\cdot j}} l^a(\Theta^{(m)})\right) \quad \forall j = 1,\ldots,p,
\end{aligned}$$

where $\mathrm{prox}_{\lambda_1,\lambda_2,\eta_m}(\cdot)$ is the proximal update. All of these optimization steps depend on the learning rate η_m at the mth iteration. We use an backtracking line search algorithm to select η_m in a data driven manner. Specifically, at each iteration m, we initialize $\eta = \eta_{m-1}$ and repeatedly shrink it by setting $\eta \leftarrow \rho\eta$ until the following condition is satisfied:

$$l^a(\Theta^+) \leq l^a(\Theta^{(m)}) + \langle \Theta^+ - \Theta^{(m)}, \nabla l^a(\Theta^{(m)}) \rangle + \frac{1}{2\eta} \|\Theta^+ - \Theta^{(m)}\|_2^2,$$

where $\Theta^+ = \mathrm{prox}_{\lambda_1,\lambda_2,\eta}\left(\Theta^{(m)} - \eta\nabla l^a(\Theta^{(m)})\right)$ is the candidate update. For a more detailed discussion on the backtracking line search algorithm, we refer the authors to Adhikari et al.[1].

References

[1] S. Adhikari, F. Lecci, J. T. Becker, B. W. Junker, L. H. Kuller, O. L. Lopez, and R. J. Tibshirani. High-dimensional longitudinal classification with the multinomial fused lasso. *Statistics in medicine*, 38(12):2184–2205, 2019.
